# 

**Published:** 1842-06

**Authors:** 


### CONTENTS.

### LIBRARY.

PAGE.

Principles of Dental Surgery; exhibiting a new method of treating the Diseases of the Teeth and Gums. By Leonard Koecker, Surgeon Dentist, London, .......................... 73

### JOURNAL.

ARTICLE I.—A Treatise on Mechanical Dentistry. By Solyman Brown, M. D., D. D. S........................ 265

ART. II.—A Dissertation on the Diseases of the Maxillary Sinuses. By W. W. H. Thackston, candidate for the degree of Doctor of Dental Surgery,........................ 279

ART. III.—Description of a case of Alveolar Abscess. By Isaac I. Greenwood, M. D., D. D. S. ........................ 291

Looseness of the Teeth,........................ 293

### BIBLIOGRAPHICAL NOTICES.

On Dentition and some coincident Disorders. By John Ashburner, M. D., Member of the Royal College of Physicians, &c. ...... 294

The Structure, Diseases, and Treatment of the Teeth considered, with a view to the abolition, in all common cases, of the pernicious practice of Tooth Drawing. By William Wardroper, M. R. C. S. S., &c........................ 297

### RECENT PUBLICATIONS.

Précis sur le redressement des dents. Par J. M. A. Schange,........ 298

### WORKS IN PROGRESS.

Institutes of Dental Surgery. By Dr. James D. M'Cabe,........... 298

A Complete Treatise on the Dental Art, based upon actual experience. By F. Maury, Dentist of the Royal Polytechnic School. Translated by Joseph B. Savier, D. D. S........... 299

### MISCELLANEOUS NOTICES.

Dr. Koecker on the Diseases of the Gums,........................ 299

Progress of Dental Surgery........................ 300

Salivation caused by fillings of the "Amalgam of Mercury and Silver" in the Teeth,........................ 301

Meeting in Boston,........................ 302

Completion of the Second Volume of the American Journal and Library of Dental Science,........................ 302

Hemorrhage from the Extraction of a Tooth,........................ 304

